# Prediction of Healthy Pregnancy Outcomes in Women with Overweight and Obesity: The Role of Maternal Early-Pregnancy Metabolites

**DOI:** 10.3390/metabo12010013

**Published:** 2021-12-24

**Authors:** Rama J. Wahab, Vincent W. V. Jaddoe, Romy Gaillard

**Affiliations:** 1 The Generation R Study Group, Erasmus MC, University Medical Center, 3000 CA Rotterdam, The Netherlands; r.wahab@erasmusmc.nl (R.J.W.); v.jaddoe@erasmusmc.nl (V.W.V.J.); 2 Department of Pediatrics, Sophia’s Children’s Hospital, Erasmus MC, University Medical Center, 3000 CA Rotterdam, The Netherlands

**Keywords:** obesity, metabolomics, pregnancy complications, obstetrics

## Abstract

Women with obesity receive intensified antenatal care due to their increased risk of pregnancy complications, even though not all of these women develop complications. We developed a model based on maternal characteristics for prediction of healthy pregnancy outcomes in women with obesity or who are overweight. We assessed whether early-pregnancy metabolites improved prediction. In a population-based cohort study among a subsample of 1180 Dutch pregnant women with obesity or who are overweight, we developed a prediction model using 32 maternal socio-demographic, lifestyle, physical and pregnancy-related characteristics. We determined early-pregnancy amino acids, nonesterifed fatty acids, phospholipids and carnitines in blood serum using liquid chromatography-tandem mass spectrometry. A healthy pregnancy outcome was the absence of fetal death, gestational hypertension, preeclampsia, gestational diabetes, caesarian section, preterm birth, large-for-gestational-age at birth, macrosomia, postpartum weight retention and offspring overweight/obesity at 5 years. Maternal age, relationship status, parity, early-pregnancy body mass index, mid-pregnancy gestational weight gain, systolic blood pressure and estimated fetal weight were selected into the model using backward selection (area under the receiver operating characteristic curve: 0.65 (95% confidence interval 0.61 to 0.68)). Early-pregnancy metabolites did not improve model performance. Thus, in women with obesity or who are overweight, maternal characteristics can moderately predict a healthy pregnancy outcome. Maternal early-pregnancy metabolites have no incremental value in the prediction of a healthy pregnancy outcome.

## 1. Introduction

Obesity among women or being overweight is currently the most common medical disorder in pregnancy [1,2,3]. Women with obesity or who are overweight during pregnancy not only have strongly increased risks of maternal and neonatal morbidity and mortality, but also of long-term adverse maternal and offspring health outcomes, including postpartum weight retention and offspring obesity [4,5]. For prevention and management of these risks, guidelines recommend intensified antenatal monitoring and care for pregnant women with obesity [6,7,8]. Nevertheless, a substantial proportion of women with obesity will have an uncomplicated pregnancy [9]. Offering these women intensified antenatal care may unnecessarily medicalize pregnancy and may not ensure the most cost-effective care. Besides, women who are overweight during pregnancy also have strongly increased risks of adverse pregnancy outcomes, and are currently not considered when appointing additional interventions during pregnancy [1]. A personalized risk assessment, using well-known risk factors associated with adverse pregnancy and long-term health outcomes could enable tailored antenatal care in these high-risk women with obesity or who are overweight during pregnancy. Previous studies have aimed to develop models for the prediction of healthy pregnancies among women with obesity, but could only achieve a moderate performance of models [9,10]. To avoid unnecessary medicalization of pregnancy, to improve pregnancy outcomes and to reduce health care burden and costs, improvement of the prediction of healthy pregnancy outcomes among women with obesity or who are overweight is needed [11,12,13]. It has been proposed that not all women with obesity or who are overweight are metabolically unhealthy [14]. Women with obesity or who are overweight who are metabolically healthy may be more prone to having a favorable pregnancy outcome [15]. Maternal early-pregnancy metabolites may offer the opportunity to distinguish the metabolically healthy from the unhealthy phenotype and could therefore provide a more accurate prediction of pregnancy complications [16,17].

In a subsample of 1180 Dutch pregnant women with obesity, or who are overweight, and their offspring from a population-based cohort study, we first developed a prediction model using maternal socio-demographic, lifestyle, physical and pregnancy-related characteristics in the first half of pregnancy to predict a healthy pregnancy outcome. Second, we assessed whether maternal early-pregnancy metabolites could improve prediction of a healthy pregnancy outcome in addition to well-known clinical risk factors. 

## 2. Results

### 2.1. Subject Characteristics

**Figure 1:**
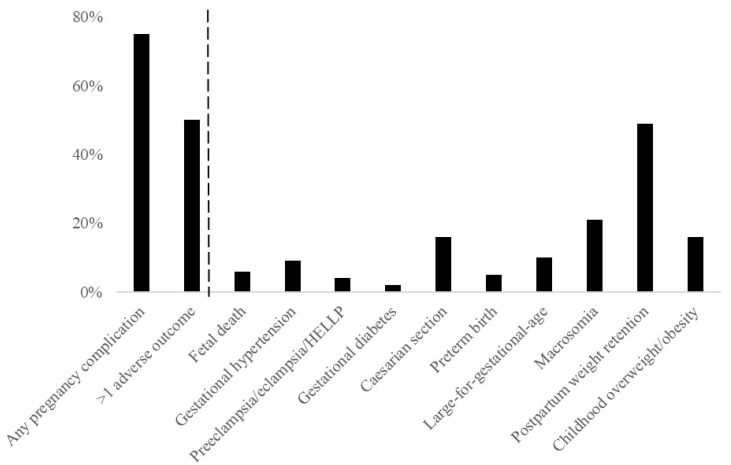
Prevalence of adverse outcomes.

Figure 1 shows that 293 (25%) of overweight and obese pregnant women had a healthy pregnancy outcome. Of women with an adverse pregnancy outcome, 447 (50%) had more than one adverse pregnancy outcome. Table 1 shows characteristics of overweight or obese women according to a healthy or adverse pregnancy outcomes. The number of women with obesity was higher among women with an adverse pregnancy outcome than among women with a healthy pregnancy outcome.

### 2.2. Model Selection

**Figure 2:**
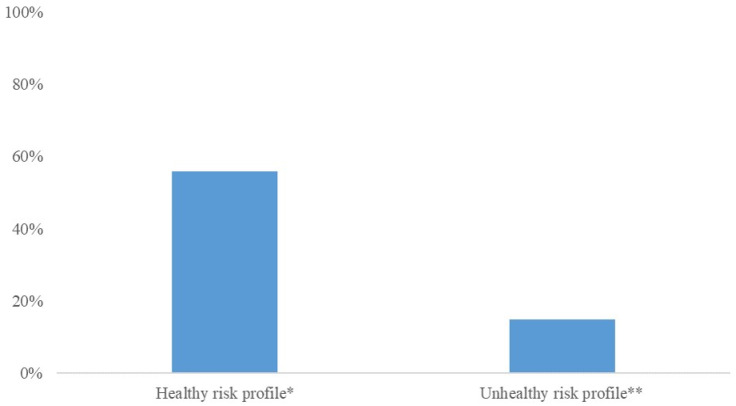
Chances of a healthy pregnancy outcome for women with different combinations of risk factors. * A health risk profile represents a 26-year-old women who is married, multiparous, a BMI of 26 kg/m^2^, 0.3 kg gestational weight gain per week, a mid-pregnancy systolic blood pressure of 110 mmHg, and a mid-pregnancy estimated fetal weight of 0.3 SDS. ** An unhealthy risk profile risk profile represents a 36-year-old women without a partner, nulliparous, a BMI of 40 kg/m^2^, 1 kg gestational weight gain per week, a mid-pregnancy blood pressure of 140 mmHg, and a mid-pregnancy estimated fetal weight of 1.5 SDS.

From the early-pregnancy maternal candidate predictors, maternal age, relationship status, parity, BMI, systolic blood pressure and CRP concentrations were selected in the model using backward selection (Table 2). This early-pregnancy model had an AUC of 0.61 (95% CI 0.58 to 0.65) with a sensitivity of 23% and positive likelihood ratio of 2.3 at 90% specificity. From the maternal mid-pregnancy candidate predictors, gestational weight gain, systolic blood pressure and estimated fetal weight in mid-pregnancy were selected in the model. The addition of these mid-pregnancy characteristics resulted in maternal early-pregnancy systolic blood pressure being removed from the model. The full model, including early-, and mid-pregnancy characteristics, had an AUC of 0.65 (95% CI 0.61; 0.68) with a sensitivity of 23% and positive likelihood ratio of 2.3 at 90% specificity (*p* = 0.016 in comparison to the early-pregnancy model). Effect estimates for the selected risk factors are shown in Table 3. A pregnant woman with obesity or who is overweight and with a healthy risk profile has a 56% chance of a healthy pregnancy outcome, whereas a women with an unhealthy risk profile has a 15% chance of a healthy pregnancy outcome (Figure 2). 

### 2.3. Maternal Metabolites

Women in the subpopulation with maternal early-pregnancy metabolite concentrations available had largely similar characteristics as women in the total population (Supplementary Table S1). From the maternal early-pregnancy metabolites, Arginine, non-esterified fatty acids (NEFA).14.0, NEFA.14.1, NEFA.16.0, NEFA.17.1 and NEFA.20.3 were selected (Table 4). The addition of these metabolites to the full maternal model did not improve the model performance (AUC: 0.70 (95% CI 0.63; 0.78) when compared to the performance of the full maternal model when assessed in the subgroup of women with metabolite concentrations available (AUC of the full maternal model in this subgroup 0.69 (95% CI 0.61; 0.76)). 

### 2.4. Sensitivity Analyses

Model selection and performance was similar to the full maternal model when only including women with early-pregnancy obesity or overweight conditions and not those only with pre-pregnancy BMI available (n population for analysis = 1027) or when excluding women with fetal deaths (n population for analysis = 11,173) (Supplementary Table S2). Among women with full information on the composite healthy pregnancy outcome available (n population for analysis = 948), model selection for a healthy pregnancy outcome was similar, but the model performance was slightly lower than in the main analyses (AUC 0.60 (95% CI 0.54; 0.67)) (Supplementary Table S2). When not considering long-term complications as an adverse pregnancy outcome, 644 (55%) of women had a healthy pregnancy outcome (n population for analysis = 1180). The model selection for the prediction of a healthy pregnancy outcome excluding postpartum weight gain and offspring with obesity or being overweight was slightly different and included maternal early-pregnancy age, BMI, vegetable consumption, protein consumption, systolic blood pressure, glucose concentrations, early-pregnancy placental growth factor (PlGF) and maternal mid-pregnancy gestational weight gain, diastolic blood pressure, PlGF concentrations, sFlt-1 concentrations and estimated fetal weight. Model performance was largely similar to the main analyses (AUC: 0.67 (95% CI 0.64; 0.70)) (Supplementary Table S2). Model selection for maternal pregnancy outcomes only included maternal age, educational level, parity, fruit consumption, early-pregnancy systolic blood pressure, CRP concentrations, gestational weight gain, mid-pregnancy PlGF concentrations and uterine artery resistance index. The model performance was similar to the main analyses (AUC: 0.65 (95% CI 0.62; 0.68)) (Supplementary Table S2). The model selection for offspring pregnancy outcomes included relationship status, parity, BMI, vegetable consumption, kcal consumption, fat consumption, protein consumption, carbohydrate consumption, HDL-concentrations, early-pregnancy sFlt-1 concentrations, gestational weight gain, mid-pregnancy PlGF concentrations, 25(OH)D concentrations and estimated fetal weight. The model performance was largely similar to the main analyses (AUC: 0.67 (95% CI 0.64; 0.70)) (Supplementary Table S2). The addition of the selected maternal early-pregnancy metabolites to this model excluding long-term outcomes of pregnancy did not improve the model performance when compared to the performance of the model when assessed in the subgroup of women with metabolite concentrations available (results not shown).

## 3. Discussion

In this population-based cohort study, a model based on common maternal socio-demographic, lifestyle, physical and pregnancy-related characteristics in the first half of pregnancy could moderately predict a healthy pregnancy. Maternal early-pregnancy metabolites had no incremental value in the prediction of a healthy pregnancy outcome. 

### 3.1. Interpretation of Main Findings

Women with obesity during pregnancy are offered uniform intensified antenatal care, due to their increased risk of pregnancy complications and long-term adverse maternal and offspring health outcomes. National and international guidelines on being overweight or obese during pregnancy do not provide sufficient targeted recommendations for tailored antenatal care in these high-risk pregnant women [8,18,19]. Accurate identification of women with obesity or who are overweight who will have a healthy pregnancy outcome is needed to enable a personalized approach of antenatal care [11,12,13].

Few studies have developed prediction models for a healthy pregnancy outcome in women with obesity during pregnancy [9,10]. A study from the United Kingdom among 1409 obese pregnant women developed a model including maternal age, parity, systolic blood pressure, HbA1c and plasma adiponectin for the prediction of a healthy pregnancy outcome (AUC of 0.72) [10]. In this study, 36% of obese pregnant women had a healthy pregnancy outcome, defined as delivery of a term live-born newborn without antenatal or labour complications. In a Canadian study among 38,055 obese pregnant women, 58% had a healthy pregnancy outcome, defined as delivery of a term live-born newborn without antenatal or labour complications [9]. Their model included maternal parity, age, income, ethnicity, weight, placenta-associated plasma protein-A and spontaneously conceived pregnancies and had an AUC of 0.58 for prediction of a healthy pregnancy outcome. 

In the current study, we observed that 25% of women with overweight and obesity had a healthy pregnancy outcome, which not only comprised short-term maternal and neonatal outcomes, but also included long-term health outcomes. Maternal gestational obesity are major risk factors for maternal postpartum weight gain and offspring obesity, which are strongly related to maternal and offspring cardio-metabolic diseases in later life [4,20]. These outcomes are crucial to take into account in the risk assessment of pregnancy outcomes among overweight and obese pregnant women. We identified maternal early-pregnancy age, relationship status, parity and BMI and mid-pregnancy gestational weight gain, systolic blood pressure and estimated fetal weight as predictors of a healthy pregnancy outcome in women with obesity or who are overweight during pregnancy. Mid-pregnancy characteristics significantly improved model performance. The effect estimate of maternal relationship status may suggest a lower risk of a healthy pregnancy outcome when being in a relationship. This finding is in contrast with our prior hypothesis, which may be due to the relatively low number of women without a relationship within our study population. Studies among larger populations including external validation samples need to further assess the role of maternal relationship status in prediction models for a healthy pregnancy outcome among women with obesity or who are overweight. In line with previous studies, our developed model could moderately predict a healthy pregnancy outcome. When focusing on short-term pregnancy outcomes and maternal and offspring pregnancy outcomes separately, model selection was slightly different. Although model performances were largely similar to the main model, differences in model selection may suggest that the prediction of a healthy pregnancy outcome in women with obesity or who are overweight may be more accurate when assessed for specific outcomes separately. In addition to previous studies focusing on women with obesity, we also included women who are overweight during pregnancy. Risks of adverse outcomes seem to be already present among pregnant women with a BMI below the threshold of obesity, which was confirmed by the additional predictive value of maternal BMI continuously in our model. Thus, our findings suggest that, in pregnant women with obesity or who are overweight, moderate prediction of a health pregnancy outcome can be achieved when using common maternal characteristics in early-, and mid-pregnancy.

It remains a major challenge to develop models that accurately predict healthy pregnancy outcomes in women with obesity or who are overweight. There is a need for identification of novel markers to discriminate women with obesity or who are overweight who will develop adverse pregnancy outcomes from those who will not. Maternal metabolite concentrations during pregnancy have become a relatively new focus for assessing maternal metabolism during pregnancy. In a previous study, maternal mid-pregnancy metabolite concentrations improved prediction pregnancy complications on top of common maternal risk factors in normal-weight women, but not in women with obesity [17]. In contrast to findings in previous studies, in the current study, branched chain amino acids (BCAA) were not selected in the model [21,22]. As BCAA reflect insulin resistance, this could be due to our relatively healthy population, with low mean glucose concentrations. It could also be due to our non-fasting samples and the timing in early pregnancy, as insulin resistance may be more pronounced in the fasting state and later in pregnancy [23,24]. The selection of NEFA metabolites into the model underlines the importance of lipid metabolism in the development of pregnancy complications in women with obesity or who are overweight. Arginine was also selected into the model. Multiple studies have shown that arginine supplementation may be useful for the prevention of pregnancy complications [25,26,27,28,29,30]. However, in the current study we did not observe an incremental value of maternal metabolites for the prediction of a healthy pregnancy outcome in addition to well-known clinical predictors. As it seems likely that all women who are overweight or obese show subclinical metabolic disruptions during pregnancy, the discriminative power of metabolites for a healthy pregnancy outcome in this high-risk population may be limited [4,5]. Thus, maternal early-pregnancy metabolite concentrations do not seem to have an incremental value in the prediction of healthy pregnancy outcomes in women with obesity or who are overweight. 

Our findings, together with findings from previous studies, indicate that a substantial proportion of women with obesity or who are overweight during pregnancy do not experience complications. This underlines that stratifying women as high risk only based on having overweight or obesity may be too simplistic. Our developed model was not sufficient to accurately predict a healthy pregnancy outcome. Especially when a prediction model should identify women who can be excluded from intensified antenatal care, a high sensitivity to eliminate possibilities of false negatives is important. Results from our study, together with results from previous studies, show that highly accurate risk stratification cannot be achieved using common characteristics and less established maternal metabolomics characteristics. Studies identifying novel markers that improve prediction of healthy pregnancy outcomes in women who are overweight or obese are urgently needed. 

### 3.2. Methodological Considerations

The major strength of this study is the prospective data collection providing a large amount of high-quality data on socio-demographic, lifestyle, physical and pregnancy-related characteristics. Multiple factors were repeatedly available in early- and mid-pregnancy to enable identification of most valuable periods for prediction of a healthy pregnancy outcome. We selected an ethnic homogeneous population, only including Dutch women, to eliminate potential statistical noise or effect modification in predictor selection by ethnicity. This selected population may have led to reduced statistical power and could have affected generalizability. Although model performance was largely similar in women with a non-Dutch ethnicity within our cohort, future models should be developed and validated to meet generalizability to multi-ethnic populations. Our relatively low number of cases of gestational diabetes and gestational hypertensive disorders in the study population suggests a selection to a relatively healthy population, which may affect the generalizability of our findings. The low number of gestational diabetes could also be due to missed diagnosis as in clinical practice there is only selective screening for gestational diabetes in women at increased risk. Further studies are needed to replicate our findings. These studies need to be conducted among populations including a sample of women with pregnancy complications corresponding with the prevalence of pregnancy complications within in the general population and should include screening for pregnancy complications among the whole study sample. We had maternal biomarker characteristics available from non-fasting blood samples. Although the use of non-fasting samples are easier to adopt in clinical settings, further studies need to assess whether the use fasting blood biomarker measurements may improve predictive performance of models. Due to the design of the study, we only had information available of maternal weight six years after birth. Events occurring in this relatively large time window, such as intervening pregnancies, may have influenced our weight measurement. We used validated questionnaires to assess socio-demographic and lifestyle characteristics, but the use of questionnaires may still induce information bias.

## 4. Materials and Methods

### 4.1. Subjects

This study was embedded in a subgroup of the Generation R Study, a population-based prospective cohort study from fetal life until adulthood in Rotterdam, the Netherlands [31]. Study approval was obtained by the Medical Ethical Committee of the Erasmus Medical Center, University Medical Center, Rotterdam (MEC 198.782/2001/31). In total, 8879 women were enrolled during pregnancy. Written informed consent was obtained from all participants. For the current study, we only included women with a Dutch ethnicity, due to the availability of metabolomics data in a preselected subsample of Dutch women. Of Dutch women, 1200 had pre-pregnancy or early-pregnancy obesity or were overweight. After exclusion of non-singleton pregnancies, induced abortions and women without information on birth characteristics, our population for analyses consisted of 1180 women (Supplementary Figure S1). For metabolomics analysis, early-pregnancy metabolites were available in a subsample of 273 of the women [32]. This subsample is a random group of mothers and their children of Dutch ethnicity selected for additional measurements within the Generation R study, already at the start of our cohort study. 

### 4.2. Maternal Clinical Candidate Predictors

For development of prediction models, we included characteristics well-known to be associated with the risk of adverse pregnancy outcomes and clustered them based on their assessment in early- or mid-pregnancy [2,3,4]. 

Early-pregnancy cluster: Information on age, ethnicity, educational level, income, relationship status, parity, history of obstetric complications, folic acid supplementation and smoking during pregnancy was obtained from questionnaires [31]. History of obstetric complications included stillbirth, miscarriage, pre-eclampsia, gestational hypertensive disorders, gestational diabetes, caesarian section, low birth weight, macrosomia. Dietary intake was assessed by a Food Frequency Questionnaire [33]. At a median gestational age of 12.9 (95% range 9.6; 17.3) weeks, we measured height, weight and blood pressure at the research center [31]. BMI (kg/m^2^) was calculated [34]. Non-fasting venous blood samples were sampled to analyze glucose (mmol/L), triglyceride (mmol/L), High Density Lipoprotein-cholesterol (HDL-cholesterol) (mmol/L) and C-reactive protein (CRP) (mg/L), Soluble fms-like tyrosine kinase 1 (sFlt-1) and placental growth factor (PlGF) concentrations. Detailed procedures for biomarker analyses are described elsewhere [31,35,36,37,38,39]. CRP concentrations >10 mg/L were excluded to eliminate high CRP concentrations due to acute infections. 

Mid-pregnancy cluster: At a median gestational age of 20.6 (95% range 18.7; 23.3) weeks, weight was measured and mid-pregnancy weight gain in kilograms/week was calculated as mid-pregnancy weight-early-pregnancy/difference in gestational age. We measured mid-pregnancy systolic and diastolic blood pressure and obtained 25(OH)D, sFlt-1 and PlGF concentrations from sampled venous blood samples [35]. Using ultrasound examinations we obtained estimated fetal weight, umbilical artery pulsatility index and uterine artery resistance index as described previously [40,41,42]. Gestational-age adjusted standard deviation-scores for estimated fetal weight ware based on reference growth charts from the whole study population and represent the equivalent of z-scores. 

### 4.3. Maternal Metabolites 

Metabolomics analysis was done in the same venous blood samples as used for clinical biomarker analyses, as was detailed previously [43]. A targeted metabolomics approach was adopted to determine serum concentrations (µmol/L) of AA, NEFA, PL and Carn [23]. We used a targeted metabolomics approach to enable assessment of absolute metabolite concentrations of metabolites. Selected maternal early-pregnancy metabolites were known a priori to be relevant for obesity and cardio-metabolic disease [22,44,45]. Associations of maternal BMI and maternal early-pregnancy metabolites within this study population are shown in Supplementary Table S3. AA were analyzed with 1100 high-performance liquid chromatography (HPLC) system (Agilent, Waldbronn, Germany) coupled to a API2000 tandem mass spectrometer (AB Sciex, Darmstadt, Germany) [46]. IUPAC-IUB Nomenclature was used for notation of AA [47]. NEFA, PL and Carn were measured with a 1200 SL HPLC system (Agilent, Waldbronn, Germany) coupled to a 4000QTRAP tandem mass spectrometer from AB Sciex (Darmstadt, Germany) [48,49]. The analytical technique used is capable of determining the total number of total bonds, but not the position of the double bonds and the distribution of the carbon atoms between fatty acid side chains. We used the following notation for NEFA, PL and Carn.a:X:Y, where X denotes the length of the carbon chain, and Y the number of double bonds. The ‘a’ denotes an acyl chain bound to the backbone via an ester bond (‘acyl-’) and the ‘e’ represents an ether bond (‘alkyl-’). For analyses, we categorized metabolites in to general metabolite groups based on chemical structure (AA, NEFA, PC.aa, PC.ae, Lyso.PC.a, Lyso.PC.e, SM, Free Carn and Carn.a). Descriptive information on maternal early-pregnancy metabolites is provided in Supplementary Table S4.

### 4.4. Healthy Pregnancy Outcome

A healthy pregnancy outcome was defined as the absence of the following outcomes: intrauterine fetal death, gestational hypertension, pre-eclampsia, gestational diabetes, caesarian section, preterm birth, large-for-gestational-age (LGA) at birth, macrosomia, maternal postpartum weight retention and offspring obesity or overweight conditions. 

Information on maternal pregnancy complications was obtained from medical records [31]. Preterm birth was defined as gestational age at birth <37 weeks. LGA was the highest ten percentiles of gestational age- and sex-adjusted birthweight [50]. Macrosomia was birthweight >4000 g. At a visit to the research center when the child reached 6 years of age, we measured maternal weight at 6 years postpartum. Maternal postpartum weight gain was defined as having a postpartum weight higher than the pre-pregnancy weight, calculated as maternal weight 6 years postpartum–maternal pre-pregnancy weight. Child’s height and weight were measured and BMI was calculated (*n* = 891). If child’s BMI at 6 years was missing, we used the last growth measured at the Community Child Health Centers (median age 3.8, 95% range 2.0; 4.0) (*n* = 144) [48]. We categorized childhood weight status in underweight/normal weight and overweight/obesity. If information on an outcome was missing, it was considered as a non-adverse outcome (number (%) of missing values per outcome: intrauterine fetal death: 0; gestational hypertension/preeclampsia: 35 (3); gestational diabetes: 43 (3.6); caesarian section: 73 (6.2); preterm birth. 

### 4.5. Statistical Analysis

Nominal and non-linear candidate predictors were categorized into clinical categories or quintiles and one missing category to allow for missing values when using the final model in clinical setting. Linear candidate predictors were used continuously and imputed with mean or median. In case of missing values in clinical practice, the mean or median can be inserted. For model selection, we used two multivariable logistic regression models to evaluate whether accurate prediction could already be performed in early-pregnancy or required mid-pregnancy characteristics. We started only selecting characteristics from the early-pregnancy cluster using backward selection and stopped when all *p*-values < 0.20. After selection from the early-pregnancy cluster, we assessed model performance by assessing the area under the receiver operating curve (AUC), sensitivity and positive likelihood ratio at 70, 80 and 90% specificity. We extended this model by including the cluster of mid-pregnancy characteristics. Based on the log-likelihood ratio, we evaluated whether the cluster of mid-pregnancy characteristics improved the model and further selected variables from this cluster using a similar approach. After model selection, we again assessed model performance of the full maternal model and compared AUCs of the early-pregnancy and the full model using DeLong test [51].

After model selection, we assessed the incremental predictive value of maternal metabolites. Due to their less established associations with a healthy pregnancy outcome, we used forward selection to select these candidate predictors (threshold *p*-value < 0.20). The cluster of selected maternal metabolites was separately added to the full maternal model, and we compared the model performance.

We performed several sensitivity analyses: (1) only including women with measured early-pregnancy obesity or being overweight, (2) only including women with full information on all adverse outcomes available, (3) excluding fetal deaths from the composite healthy pregnancy outcome, (4) excluding postpartum weight retention and childhood obesity or an overweight condition from the composite healthy pregnancy outcome and (5) seperating for maternal pregnancy outcomes (gestational hypertensive disorders, preeclampsia, gestational diabetes, caesarian section and postpartum weight retention) and offspring pregnancy outcomes (intrauterine fetal death, preterm birth, large-for-gestational-age at birth, macrosomia and offspring obesity or being overweight). If different predictors were selected into the models, we additionally tested the additional predictive value of maternal early-pregnancy metabolites for those models. The statistical analyses were performed using the Statistical Package of Social Sciences version 24.0 for Windows (SPSS Inc., Chicago, IL, USA).

## 5. Conclusions

Common maternal socio-demographic, lifestyle, physical and pregnancy-related characteristics in the first half of pregnancy can moderately predict a healthy pregnancy outcome in women with obesity or who are overweight. Maternal early-pregnancy metabolites do not improve prediction of a healthy pregnancy outcome. Future studies need to identify novel markers to achieve accurate prediction of healthy pregnancies in overweight and obese pregnant women to enable tailored antenatal care within this high-risk population.

## Figures and Tables

**Table 1:** Population characteristics.

	Total Group (*n* = 1180)	Healthy Pregnancy Outcome (*n* = 293)	Any Adverse Pregnancy Outcome (*n* = 887)
Early-pregnancy characteristics			
Gestational age at measurement, median (95% range), weeks	12.9 (9.6; 17.3)	12.9 (8.2; 17.2)	12.9 (9.8; 17.4)
Age, mean (SD), years	31.1 (4.4)	30.2 (4.5)	31.3 (4.4)
Prepregnancy Body Mass Index, median (95% range), kg/m^2^	26.6 (23.0; 38.1)	26.5 (23.4; 36.7)	26.7 (23.0; 38.5)
Prepregnancy obesity, n yes (%)	228 (23)	42 (19)	186 (24)
Early-pregnancy Body Mass Index, median (95% range), kg/m^2^	27.6 (25.0; 38.6)	27.7 (25.1; 38.9)	27.7 (25.1; 38.9)
Early-pregnancy obesity, n yes (%)	297 (28)	63 (24)	234 (30)
Parity, n multiparous (%)	518 (44)	140 (48)	378 (43)
Education, n higher education (%)	522 (45)	119 (41)	403 (46)
Income, n > 2200 euro (%)	714 (71)	167 (68)	547 (71)
Relationship status, n married or living together (%)	1070 (94)	254 (90)	816 (95)
History of obstetric complications, n no (%)	392 (97)	97 (97)	295 (97)
Smoking, n no (%)	779 (72)	185 (70)	594 (72)
Folic acid supplementation, n yes (%)	837 (86)	190 (83)	647 (88)
Fruit consumption, n ≥ 200 grams/day, n yes (%)	638 (54)	164 (64)	474 (61)
Vegetable consumption, n ≥ 250 grams/day, n yes (%)	67 (6)	18 (7)	49 (6)
Energy intake, mean (SD), kcal/day	2090 (508)	2062 (517)	2101 (505)
Carbohydrate intake, mean (SD), g/day	256 (75)	252 (78)	257 (74)
Fat intake, mean (SD), g/day	84 (24)	83 (23)	84 (24)
Protein intake, mean (SD), g/day	77 (19)	76 (20)	78 (19)
Systolic blood pressure, mean (SD), mmHg	123 (13)	122 (13)	122 (13)
Diastolic blood pressure, mean (SD), mmHg	73.1 (9.9)	72 (10)	73 (10)
Glucose, mean (SD), mmol/L	4.5 (0.9)	4.4 (0.7)	4.5 (0.9)
HDL-concentrations, mean (SD), mmol/L	1.7 (0.3)	1.7 (0.3)	1.7 (0.3)
Triglycerides concentrations, median (95% range), mmol/L	1.4 (0.7; 2.8)	1.4 (0.7; 2.7)	1.4 (0.7; 2.8)
CRP concentrations, median (95% range), mg/L	4.9 (0.9; 9.6)	5.2 (0.8; 9.7)	4.8 (0.9; 9.6)
Placental growth factor, median (95% range), mom	0.99 (0.42; 4.21)	1.05 (0.39; 3.86)	0.99 (0.39; 4.31)
sFlt-1, median, (95% range), mom	1.00 (0.41; 2.60)	1.02 (0.42; 2.59)	0.99 (0.39; 2.62)
Mid-pregnancy characteristics			
Gestational age at measurement, median (95% range), weeks	20.6 (18.7; 23.3)	20.4 (18.7; 23.3)	20.5 (18.8; 23.5)
Mid-pregnancy weight, median (95% range), kg/m^2^	84.0 (69.0; 116.0)	82.0 (67.5; 112.2)	84.8 (70.0; 117.0)
Gestational weight gain, median (95% range), kg/week	0.29 (−0.19; 0.71)	0.24 (−0.24; 0.67)	0.30 (−0.15; 0.72)
Systolic blood pressure, mean (SD), mmHg	123 (12)	122 (11)	125 (13)
Diastolic blood pressure, mean (SD), mmHg	72 (10)	71 (9)	72 (10)
25(OH)D concentrations, median (95% range), nmol/L	60.1 (16.3; 121.9)	59.9 (13.5; 114.2)	60.3 (16.6; 122.7)
Placental growth factor, median (95% range), mom	1.00 (0.39; 3.15)	0.97 (0.37; 3.39)	1.01 (0.40; 2.93)
sFlt-1, median, (95% range), mom	1.00 (0.33; 3.15)	0.99 (0.31; 2.99)	1.00 (0.33; 3.48)
Estimated fetal weight, mean (SD), SDS	0.01 (1.00)	−0.14 (0.97)	0.05 (1.00)
Uterine artery resistance index, mean (SD), SDS	0.00 (1.00)	0.03 (0.97)	−0.01 (1.01)
Umbilical artery pulsatility index, mean (SD), SDS	0.00 (1.00)	0.08 (1.03)	−0.02 (0.99)
Birth characteristics			
Sex, n female (%)	594 (51)	146 (50)	448 (51)
Gestational age at birth, median (95%), weeks	40.3 (35.5; 42.3)	40.3 (37.1; 42.3)	40.3 (34.4; 42.3)
Birthweight, mean (SD), grams	3534 (591)	3370 (389)	3590 (635)

Percentage are valid percentages.

**Table 2:** Model selection for no adverse outcome of pregnancy.

Model Selection Based on Clusters of Maternal Clinical Candidate Predictors										
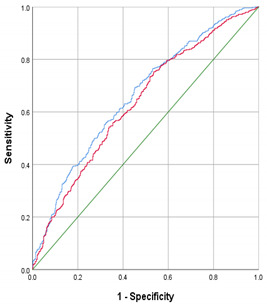	**Models**	**Variables Included per Model**	**AUC (95% CI)**	**Sensitivity at Specificity (%)**			**Positive Likelihood Ratio at Specificity**			*p*-Value *
				**70%**	**80%**	**90%**	**70%**	**80%**	**90%**	
	Early-pregnancy (red line)	Age + relationship status + parity + BMI + early-pregnancy systolic blood pressure + early-pregnancy CRP concentrations	0.61 (0.58; 0.65)	48	37	23	1.6	1.9	2.3	
	Full maternal (blue line)	Age + relationship status + parity + BMI + mid-pregnancy gestational weight gain + mid-pregnancy systolic blood pressure + mid-pregnancy estimated fetal weight	0.65 (0.61; 0.68)	50	41	23	1.7	2.1	2.3	0.016

Models were adjusted for gestational age at early-pregnancy measurement to enable interpretation of effect estimates of biomarkers not standardized. CRP: C-reactive protein. * *p*-value is obtained using DeLong’s test for comparison of the AUC of the full model with the AUC of early-pregnancy model.

**Table 3:** Effect estimates of characteristics associated with no adverse outcomes.

	Multivariable, Early-Pregnancy Model OR (95% CI) *	Multivariable, Mid-Pregnancy Model OR (95% CI) *
Early-pregnancy characteristics		
Intercept	36.80	102.26
Age (per 1 year increase)	0.94 (0.91 to 0.97)	0.94 (0.91 to 0.97)
Relationship status		
No partner	Reference	Reference
Married or in a relationship	0.60 (0.36 to 1.00)	0.58 (0.35 to 0.98)
Missing	0.98 (0.41 to 2.31)	0.88 (0.36 to 2.11)
Parity		
Nulliparous	Reference	Reference
Multiparous	1.41 (1.06 to 1.87)	1.37 (1.03 to 1.82)
BMI (per 1 kg/m^2^ increase)	0.96 (0.92 to 1.00)	0.96 (0.92 to 1.00)
Systolic blood pressure (per 10 mmHg increase)	0.91 (0.81 to 1.03)	
CRP concentrations	1.05 (0.98 to 1.13)	
Mid-pregnancy characteristics		
Gestational weight gain (per 1 kg/week increase)		0.44 (0.22 to 0.89)
Systolic blood pressure (per 10 mmHg)		0.89 (0.79 to 1.00)
Estimated fetal weight		
First quintile		1.10 (0.71 to 1.72)
Second quintile		0.82 (0.51 to 1.29)
Third quintile		Reference
Fourth quintile		0.79 (0.50 to 1.26)
Fifth quintile		0.49 (0.30 to 0.82)
Missing		1.18 (0.70 to 1.98)

* All effect estimates were adjusted for gestational age at measurement in early-pregnancy.

**Table 4:** Selection and performance of maternal metabolites.

No Adverse Outcome of Pregnancy									
Models	Variables Included per Model	AUC (95% CI)	Sensitivity at Specificity (%)			Positive Likelihood Ratio			*p*-Value *
			70%	80%	90%	70%	80%	90%	
Metabolomics (n = 273)	Full model + Arg + NEFA.14.0 + NEFA.14.1 + NEFA.16.0 + NEFA.17.1 + NEFA.20.3	0.70 (0.63; 0.78)	56	47	37	1.9	2.4	3.7	0.240

For selection of metabolites, a forward selection procedure was used, with a *p*-value threshold of <0.20. * *p*-values are obtained using DeLong’s test for comparison of the AUC of the full model with the AUC of the mid-pregnancy model. AUC of the full model was 0.69 (95% CI 0.61; 0.76) in the subsample with metabolomics available.

## Data Availability

The datasets generated during and/or analyzed during the current study are not publicly available but are available from the corresponding author on reasonable request because of it’s usage in the ongoing study.

## Supplementary Materials

The following are available online at https://www.mdpi.com/article/10.3390/metabo12010013/s1, Figure S1: Flow chart of the study participants, Table S1: Characteristics for women with metabolomics analyses available, Table S2: Sensitivity analyses for selected outcomes, Table S3: Maternal early-pregnancy metabolite.
