# Supplementary material for: Prediction of Healthy Pregnancy Outcomes in Women with Overweight and Obesity: The Role of Maternal Early-Pregnancy Metabolites
Source: Metabolites. 2021 Dec 24;12(1):13. doi: 10.3390/metabo12010013 (PMC8780068; doi:10.3390/metabo12010013)
Supplement: Supplementary file 1 [file metabolites-12-00013-s001.zip › metabolites-1507977-supplementary.pdf]

Supplementary Material

Prediction of Healthy Pregnancy Outcomes in Women with Overweight and Obesity: The Role of Maternal Early-Pregnancy Metabolites

Rama J. Wahab [1,2], Vincent W. V. Jaddoe [1,2] and Romy Gaillard [1,2,*]

[1] The Generation R Study Group, Erasmus MC, University Medical Center, 3000 CA, Rotterdam, The Netherlands; r.wahab@erasmusmc.nl (R.J.W.); v.jaddoe@erasmusmc.nl (V.W.V.J.)
[2] Department of Pediatrics, Sophia's Children's Hospital, Erasmus MC, University Medical Center, Rotterdam, The Netherlands
[*] Correspondence: r.gaillard@erasmusmc.nl; The Generation R Study Group, Erasmus University Medical Center, PO Box 2040, 3000 CA Rotterdam, The Netherlands; Tel.: 0031 10 704 3405

Figure S1. Flow chart of study participants.

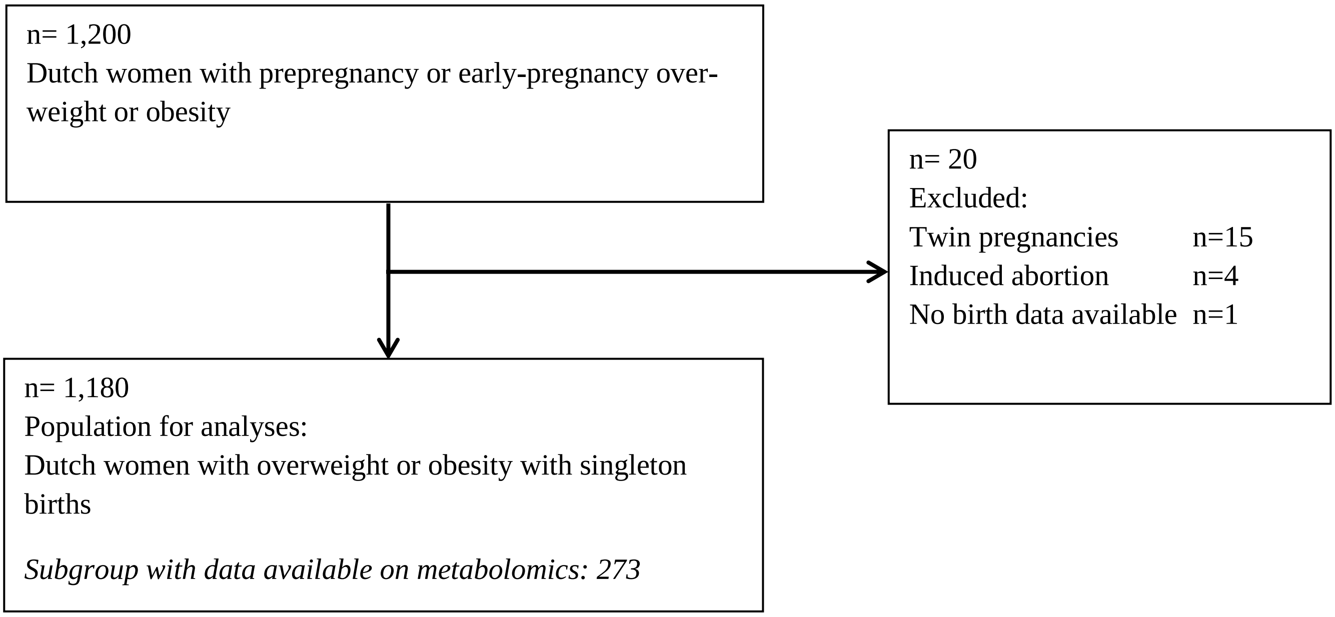

Table S1. Characteristics for women with metabolomics analyses available.

	Total group (n=1,180)	Metabolomics (n=273)
**Early-pregnancy characteristics**		
Gestational age at measurement, median (95% range), weeks	12.9 (9.6; 17.3)	12.5 (9.0; 16.8)
Age, mean (SD), years	31.1 (4.4)	31.4 (4.0)
Prepregnancy Body Mass Index, median (95% range), kg/m^2	26.6 (23.0; 38.1)	26.8 (23.2; 37.4)
Prepregnancy obesity, n yes (%)	228 (23)	50 (23)
Early-pregnancy Body Mass Index, median (95% range), kg/m^2	27.6 (25.0; 38.6)	28.8 (25.0; 37.2)
Early-pregnancy obesity, n yes (%)	297 (28)	78 (29)
Parity, n multiparous (%)	518 (44)	119 (44)
Education, n higher education (%)	522 (45)	133 (49)
Income, n >2200 euro (%)	714 (71)	195 (77)
Relationship status, n married or living together (%)	1070 (94)	253 (97)
History of obstetric complications, n no (%)	392 (97)	86 (97)
Smoking, n no (%)	779 (72)	181 (75)
Folic acid supplementation, n yes (%)	837 (86)	199 (90)
Fruit consumption, n ≥200grams/day, n yes (%)	638 (54)	149 (63)
Vegetable consumption, n ≥250grams/day, n yes (%)	67 (6)	12 (5)
Energy intake, mean (SD), kcal/day	2090 (508)	2071 (480)
Carbohydrate intake, mean (SD), g/day	256 (75)	250 (65)
Fat intake, mean (SD), g/day	84 (24)	84 (24)
Protein intake, mean (SD), g/day	77 (19)	78 (19)
Systolic blood pressure, mean (SD), mmHg	123 (13)	125 (14)
Diastolic blood pressure, mean (SD), mmHg	73.1 (9.9)	75 (11)
Glucose, mean (SD), mmol/L	4.5 (0.9)	4.5 (0.9)
HDL-concentrations, mean (SD), mmol/L	1.7 (0.3)	1.7 (0.3)
Triglycerides concentrations, median (95% range), mmol/L	1.4 (0.7; 2.8)	1.3 (0.8; 2.6)
CRP concentrations, median (95% range), mg/L	4.9 (0.9; 9.6)	5.3 (0.9; 9.7)
Placental growth factor, median (95% range), mom	0.99 (0.42; 4.21)	0.85 (0.38; 3.18)
sFlt-1, median, (95% range), mom	1.00 (0.41; 2.60)	1.02 (0.41; 2.67)
**Mid-pregnancy characteristics**		
Gestational age at measurement, median (95% range), weeks	20.6 (18.7; 23.3)	20.4 (18.9; 22.8)
Mid-pregnancy weight, median (95% range), kg/m^2	84.0 (69.0; 116.0)	84.6 (69.0; 114.6)
Gestational weight gain, median (95% range), kg/week	0.29 (-0.19; 0.71)	0.29 (-0.15; 0.70)
Systolic blood pressure, mean (SD), mmHg	123 (12)	124 (13)
Diastolic blood pressure, mean (SD), mmHg	72 (10)	73 (11)
Vitamin D concentrations, median (95% range), nmol/L	60.1 (16.3; 121.9)	61.6 (18.9; 117.7)
Placental growth factor, median (95% range), mom	1.00 (0.39; 3.15)	1.00 (0.46; 3.55)
sFlt-1, median, (95% range), mom	1.00 (0.33; 3.15)	1.01 (0.32; 3.53)
Estimated fetal weight, mean (SD), SDS	0.01 (1.00)	-0.02 (0.92)
Uterine artery resistance index, mean (SD), SDS	0.00 (1.00)	0.02 (0.94)
Umbilical artery pulsatility index, mean (SD), SDS	0.00 (1.00)	-0.05 (0.91)
**Birth characteristics**		
Sex, n female (%)	594 (51)	125 (46)
Gestational age at birth, median (95%), weeks	40.3 (35.5; 42.3)	40.4 (36.1; 42.4)
Birthweight, mean (SD), grams	3534 (591)	3588 (576)
Healthy pregnancy outcome, n yes (%)	293 (25)	70 (26)

Table S2. Sensitivity analyses for selected outcomes.

		No adverse outcome of pregnancy						
**Models**	**Variables selected**	**AUC (95% CI)**	**Sensitivity at specificity (%)**			**Positive likelihood ratio**		
			**70%**	**80%**	**90%**	**70%**	**80%**	**90%**
Only including women with early-pregnancy overweight or obesity (n=1,027)	Similar to full maternal model*	0.64 (0.60; 0.68)	49	37	22	1.6	1.9	2.2
Complete cases for pregnancy outcomes (n=948)	Similar to full maternal model	0.60 (0.54; 0.67)	38	31	12	1.3	1.6	1.2
Excluding fetal deaths (n=1,173)	Similar to full maternal model	0.65 (0.61; 0.68)	48	37	23	1.6	1.9	2.3
Excluding long-term outcomes of pregnancy (n=1,180)	Age + BMI + vegetable consumption + protein consumption + early-pregnancy systolic blood pressure + glucose concentrations + early-pregnancy PlGF concentrations + gestational weight gain + mid-pregnancy diastolic blood pressure + mid-pregnancy PlGF concentrations + mid-pregnancy sFlt-1 concentrations + estimated fetal weight	0.67 (0.64; 0.70)	52	39	23	1.7	2.0	2.3
Including only maternal pregnancy outcomes	Age + educational level + parity + vegetable consumption + fruit consumption + early-pregnancy systolic blood pressure + CRP concentrations + gestational weight gain + mid-pregnancy PlGF concentrations + UtRI	0.65 (0.62; 0.68)	52	40	26	1.7	2.0	2.6
Including only offspring pregnancy outcomes	Relationship status + parity + BMI + vegetable consumption + kcal consumption + fat consumption + protein consumption + carbohydrate consumption + HDL-concentrations + early-pregnancy sFlt-1 concentrations + gestational weight gain + mid-pregnancy PlGF concentrations + 25(OH)D concentrations + estimated fetal weight	0.67 (0.64; 0.70)	53	37	22	1.8	1.9	2.2

Models were adjusted for gestational age at early-pregnancy measurement to enable interpretation of effect estimates of biomarkers not standardized. sFlt-1: Soluble fms-like tyrosine kinase 1, CRP: C-reactive protein, PlGF: placental growth factor, UtRI: uterine artery resistance index.*The full maternal model included maternal age, relationship status, parity, BMI, mid-pregnancy gestational weight gain, mid-pregnancy systolic blood pressure and mid-pregnancy estimated fetal weight. The full maternal model had an AUC of 0.65 (95% confidence interval 0.61 to 0.68).

Table S3. Associations of maternal BMI with maternal early-pregnancy metabolites.

	Maternal BMI		
**Metabolite**	**Effect estimate**	**95% confidence interval**	**p-value**
Ala	0.049	-0.034; 0.131	0.246
Arg	0.057	0.019; 0.094	0.003
Asn	-0.018	-0.046; 0.011	0.221
Asp	0.068	0.038; 0.099	0.000
Cit	-0.011	-0.031; 0.01	0.315
Gln	-0.068	-0.151; 0.016	0.114
Glu	0.065	0.014; 0.115	0.013
Gly	0.010	-0.047; 0.068	0.722
His	-0.051	-0.095; -0.008	0.02
Ile	0.025	-0.02; 0.07	0.279
Leu	0.038	-0.019; 0.095	0.189
Lys	0.048	-0.006; 0.102	0.084
Met	-0.001	-0.024; 0.023	0.951
Orn	0.038	0.001; 0.075	0.043
Phe	0.037	0.004; 0.071	0.030
Pro	0.056	-0.023; 0.136	0.163
Trp	-0.016	-0.048; 0.017	0.339
Ser	0.013	-0.031; 0.058	0.560
Thr	0.047	-0.007; 0.101	0.090
Tyr	0.032	-0.009; 0.072	0.123
Val	0.047	-0.019; 0.113	0.164
Cys	0.012	-0.017; 0.04	0.423
NEFA_14_0	0.039	0.014; 0.065	0.003
NEFA_14_1	0.023	0.011; 0.035	0.000
NEFA_15_0	0.01	0.000; 0.000	0.059
NEFA_16_0	0.112	0.041; 0.183	0.002
NEFA_16_1	0.062	0.031; 0.093	0.000
NEFA_16_2	0.010	0.005; 0.016	0.000
NEFA_17_0	0.012	0.002; 0.023	0.025
NEFA_17_1	0.015	0.006; 0.024	0.001
NEFA_17_2	0.003	0.001; 0.006	0.013
NEFA_18_0	0.026	-0.016; 0.068	0.219
NEFA_18_1	0.118	0.038; 0.198	0.004
NEFA_18_2	0.065	0.014; 0.116	0.013
NEFA_18_3	0.02	-0.001; 0.04	0.057
NEFA_19_1	0.007	0.002; 0.013	0.01
NEFA_20_1	0.009	0.001; 0.018	0.032
NEFA_20_2	0.010	0.003; 0.017	0.006
NEFA_20_3	0.015	0.007; 0.023	0.000
NEFA_20_4	0.023	0.009; 0.038	0.002
NEFA_20_5	0.005	-0.001; 0.01	0.095
NEFA_22_3	0.003	0.001; 0.004	0.002
NEFA_22_4	0.009	0.005; 0.013	0.000
NEFA_22_5	0.007	0.001; 0.013	0.016
NEFA_22_6	0.008	-0.004; 0.021	0.196
NEFA_24_0	-0.002	-0.006; 0.002	0.355
NEFA_24_1	-0.001	-0.004; 0.002	0.711
NEFA_24_2	0.000	-0.001; 0.002	0.455
NEFA_24_4	0.001	0.000; 0.000	0.025
NEFA_24_5	0.002	0.000; 0.000	0.084
NEFA_26_0	0.000	-0.003; 0.004	0.897
NEFA_26_1	0.000	-0.002; 0.002	0.998
NEFA_26_2	0.000	-0.002; 0.002	0.921
lyso.PC.a.C14.0	0.015	0.000; 0.000	0.057
lyso.PC.a.C16.0	0.063	0.016; 0.111	0.009
lyso.PC.a.C16.1	0.018	0.008; 0.027	0.000
lyso.PC.a.C18.0	0.019	-0.005; 0.043	0.119
lyso.PC.a.C18.1	0.000	-0.021; 0.020	0.978

lyso.PC.a.C18.2	-0.023	-0.054; 0.009	0.159
lyso.PC.a.C18.3	-0.002	-0.007; 0.003	0.494
lyso.PC.a.C20.3	0.017	0.008; 0.026	0.000
lyso.PC.a.C20.4	0.021	0.007; 0.034	0.002
lyso.PC.a.C20.5	0.001	-0.004; 0.007	0.561
lyso.PC.a.C22.6	-0.006	-0.014; 0.003	0.193
lyso.PC.e.C16.0	0.001	-0.005; 0.007	0.785
lyso.PC.e.C18.0	-0.003	-0.011; 0.005	0.419
lyso.PC.e.C18.1	0.003	0.000; 0.000	0.048
PC.aa.C30.0	-0.004	-0.021; 0.014	0.684
PC.aa.C30.3	0.002	-0.001; 0.005	0.131
PC.aa.C32.0	0.015	-0.002; 0.032	0.083
PC.aa.C32.1	0.034	0.002; 0.065	0.037
PC.aa.C32.2	0.004	-0.016; 0.023	0.716
PC.aa.C32.3	0.006	0.002; 0.010	0.005
PC.aa.C34.1	0.066	-0.019; 0.151	0.126
PC.aa.C34.2	0.07	-0.044; 0.185	0.229
PC.aa.C34.3	0.01	-0.019; 0.040	0.48
PC.aa.C34.4	0.009	-0.003; 0.021	0.151
PC.aa.C34.5	-0.001	-0.004; 0.003	0.715
PC.aa.C36.0	0.001	-0.007; 0.009	0.841
PC.aa.C36.1	0.014	-0.022; 0.051	0.439
PC.aa.C36.2	0.015	-0.063; 0.093	0.702
PC.aa.C36.3	0.096	0.019; 0.173	0.014
PC.aa.C36.4	0.124	0.044; 0.204	0.002
PC.aa.C36.5	0.020	-0.015; 0.054	0.263
PC.aa.C36.6	-0.003	-0.012; 0.005	0.465
PC.aa.C38.0	0.009	-0.001; 0.019	0.09
PC.aa.C38.2	0.013	-0.002; 0.028	0.097
PC.aa.C38.3	0.083	0.038; 0.128	0.000
PC.aa.C38.4	0.085	0.033; 0.138	0.001
PC.aa.C38.5	0.023	-0.017; 0.063	0.251
PC.aa.C38.6	-0.011	-0.072; 0.049	0.711
PC.aa.C40.0	0.002	-0.005; 0.009	0.616
PC.aa.C40.1	-0.001	-0.006; 0.005	0.833
PC.aa.C40.2	0.001	-0.002; 0.005	0.542
PC.aa.C40.3	-0.001	-0.006; 0.004	0.744
PC.aa.C40.4	0.017	0.005; 0.029	0.005
PC.aa.C40.5	0.005	-0.015; 0.026	0.626
PC.aa.C40.6	0.019	-0.014; 0.052	0.252
PC.aa.C42.0	0.003	-0.002; 0.009	0.208
PC.aa.C42.5	-0.003	-0.007; 0.001	0.168
PC.aa.C43.6	0.003	-0.007; 0.013	0.554
PC.aa.C44.12	0.001	-0.004; 0.007	0.571
PC.ae.C30.0	-0.001	-0.006; 0.003	0.568
PC.ae.C32.0	0.004	-0.006; 0.013	0.420
PC.ae.C32.1	0.002	-0.006; 0.011	0.559
PC.ae.C32.2	0.001	-0.003; 0.006	0.538
PC.ae.C34.0	-0.001	-0.008; 0.006	0.767
PC.ae.C34.1	0.004	-0.011; 0.020	0.583
PC.ae.C34.2	0.001	-0.017; 0.018	0.925
PC.ae.C34.3	-0.001	-0.018; 0.016	0.913
PC.ae.C34.4	-0.001	-0.004; 0.003	0.679
PC.ae.C36.0	0.000	-0.004; 0.005	0.893
PC.ae.C36.1	-0.004	-0.017; 0.009	0.524
PC.ae.C36.2	-0.011	-0.032; 0.01	0.308
PC.ae.C36.3	0.008	-0.008; 0.025	0.324
PC.ae.C36.4	0.028	0.009; 0.048	0.005
PC.ae.C36.5	0.022	0.005; 0.039	0.013
PC.ae.C38.0	0.000	-0.011; 0.011	0.982
PC.ae.C38.2	-0.004	-0.012; 0.004	0.309
PC.ae.C38.3	0.003	-0.009; 0.014	0.613

PC.ae.C38.4	0.013	-0.004; 0.030	0.136
PC.ae.C38.5	0.032	0.011; 0.052	0.002
PC.ae.C38.6	0.014	-0.001; 0.029	0.064
PC.ae.C40.0	0.004	-0.021; 0.030	0.739
PC.ae.C40.1	0.001	-0.007; 0.008	0.86
PC.ae.C40.2	0.001	-0.007; 0.009	0.781
PC.ae.C40.3	-0.002	-0.008; 0.004	0.602
PC.ae.C40.4	0.003	-0.005; 0.011	0.497
PC.ae.C40.5	0.004	-0.005; 0.013	0.388
PC.ae.C40.6	-0.004	-0.015; 0.007	0.513
PC.ae.C42.1	0.001	-0.003; 0.005	0.538
PC.ae.C42.3	-0.002	-0.008; 0.004	0.526
PC.ae.C42.4	0.000	-0.006; 0.006	0.940
PC.ae.C42.5	0.002	-0.008; 0.013	0.646
PC.ae.C42.6	0.000	-0.007; 0.008	0.901
SM.a.C30.1	0.002	-0.002; 0.006	0.359
SM.a.C32.1	0.006	-0.008; 0.019	0.418
SM.a.C32.2	0.010	0.005; 0.015	0.000
SM.a.C33.1	0.001	-0.01; 0.011	0.921
SM.a.C34.1	0.051	0.012; 0.09	0.01
SM.a.C34.2	0.048	0.03; 0.067	0.000
SM.a.C35.0	0.006	0.002; 0.011	0.008
SM.a.C35.1	0.002	-0.006; 0.01	0.616
SM.a.C36.1	0.038	0.018; 0.057	0.000
SM.a.C36.2	0.042	0.026; 0.058	0.000
SM.a.C36.3	0.014	0.008; 0.019	0.000
SM.a.C37.1	0.007	-0.001; 0.015	0.097
SM.a.C38.2	0.023	-0.003; 0.05	0.08
SM.a.C38.3	0.005	-0.001; 0.01	0.079
SM.a.C39.1	0.000	-0.012; 0.013	0.95
SM.a.C39.2	0.008	0.001; 0.015	0.034
SM.a.C40.2	0.026	-0.003; 0.055	0.074
SM.a.C40.5	0.006	-0.002; 0.013	0.14
SM.a.C41.1	0.022	0.004; 0.04	0.017
SM.a.C41.2	0.013	-0.004; 0.031	0.132
SM.a.C42.1	0.027	0.007; 0.048	0.009
SM.a.C42.2	0.054	0.019; 0.088	0.002
SM.a.C42.3	0.04	0.015; 0.065	0.002
SM.a.C42.4	0.024	0.008; 0.039	0.003
SM.a.C42.6	0.002	-0.011; 0.014	0.764
SM.a.C43.1	0.007	0.002; 0.013	0.009
SM.a.C43.2	0.008	-0.001; 0.018	0.067
SM.a.C44.6	0.003	-0.005; 0.01	0.466
SM.e.C36.2	0.005	0.002; 0.009	0.003
SM.e.C38.3	0.002	-0.002; 0.006	0.329
SM.e.C40.5	0.004	0.000; 0.000	0.038
Carn	0.023	0.002; 0.043	0.032
Carn.a.C10.0	0.005	0.002; 0.009	0.005
Carn.a.C10.1	0.004	0.002; 0.006	0.000
Carn.a.C12.0	0.003	00;00.0	0.016
Carn.a.C14.1	0.003	0.001; 0.005	0.003
Carn.a.C14.2	0.001	0.000; 0.000	0.052
Carn.a.C15.0	0.002	0.000; 0.000	0.043
Carn.a.C16.0	0.003	0.000; 0.000	0.047
Carn.a.C16.0.Oxo	0.000	-0.001; 0.002	0.524
Carn.a.C16.1	0.002	0.000; 0.000	0.033
Carn.a.C16.2	0.001	0.000; 0.000	0.150
Carn.a.C18.0	0.002	-0.001; 0.004	0.141
Carn.a.C18.1	0.003	0.001; 0.005	0.002
Carn.a.C18.2	0.001	0.000; 0.000	0.033
Carn.a.C18.2.OH	0.001	0.000; 0.000	0.044
Carn.a.C2.0	0.013	0.004; 0.022	0.007

Carn.a.C20.0	0.002	0.001; 0.003	0.004
Carn.a.C20.1	0.000	0.000; 0.000	0.15
Carn.a.C20.3	0.001	-0.001; 0.003	0.337
Carn.a.C20.4	0.000	0.000; 0.000	0.177
Carn.a.C3.0	0.002	-0.001; 0.004	0.205
Carn.a.C3.0.DC	0.001	-0.001; 0.003	0.194
Carn.a.C4.0	0.001	-0.002; 0.004	0.528
Carn.a.C5.0	0.002	0.000; 0.000	0.117
Carn.a.C6.0	0.002	0.001; 0.003	0.000
Carn.a.C6.0.OH	0.001	0.000; 0.000	0.191
Carn.a.C8.0	0.003	0.000; 0.000	0.066
Carn.a.C8.1	0.003	0.002; 0.005	0.000
Carn.a.C9.0	0.001	-0.001; 0.002	0.350

Table S4. Maternal early-pregnancy metabolites.

Metabolite	N	Median (95% range), µmmol/L
Ala	273	428.00 (268.98; 653.41)
Arg	273	95.49 (59.70; 147.98)
Asn	273	62.35 (42.19; 89.91)
Asp	273	40.90 (23.59; 67.55)
Cit	273	19.67 (12.16; 33.59)
Gln	273	440.61 (292.27; 742.32)
Glu	273	97.13 (50.28; 161.88)
Gly	273	221.97 (136.65; 330.31)
His	273	102.36 (58.42; 162.67)
Ile	273	62.25 (30.90; 116.56)
Leu	273	132.21 (75.55; 237.83)
Lys	273	167.48 (103.05; 265.54)
Met	273	22.68 (13.27; 39.58)
Orn	273	59.51 (33.10; 100.38)
Phe	273	90.64 (57.79; 131.53)
Pro	273	198.30 (108.80; 407.19)
Trp	273	59.93 (38.27; 98.50)
Ser	273	112.41 (61.00; 181.66)
Thr	273	144.07 (91.16; 243.87)
Tyr	273	64.83 (37.75; 115.47)
Val	273	245.76 (151.13; 381.72)
Cys	273	16.27 (5.94; 31.34)
NEFA_14_0	273	4.21 (1.14; 15.41)
NEFA_14_1	273	0.62 (0.13; 2.98)
NEFA_15_0	273	0.86 (0.23; 2.92)
NEFA_16_0	273	43.20 (12.89; 136.91)
NEFA_16_1	273	4.67 (1.54; 20.47)
NEFA_16_2	273	0.17 (0.03; 0.65)
NEFA_17_0	273	0.94 (0.27; 3.01)
NEFA_17_1	273	0.44 (0.07; 1.64)
NEFA_17_2	273	0.02 (0.00; 0.10)
NEFA_18_0	273	10.92 (1.57; 34.96)
NEFA_18_1	273	43.73 (14.73; 163.22)
NEFA_18_2	273	24.46 (9.66; 81.23)
NEFA_18_3	273	2.59 (0.79; 9.70)
NEFA_19_1	273	0.21 (0.06; 0.74)
NEFA_20_1	273	0.64 (0.18; 2.01)
NEFA_20_2	273	0.45 (0.15; 1.42)
NEFA_20_3	273	0.76 (0.23; 2.01)
NEFA_20_4	273	4.55 (1.92; 9.78)
NEFA_20_5	273	0.27 (0.10; 0.86)
NEFA_22_3	273	0.03 (0.01; 0.09)
NEFA_22_4	273	0.30 (0.14; 0.75)
NEFA_22_5	273	0.43 (0.18; 1.21)
NEFA_22_6	273	1.44 (0.45; 4.66)
NEFA_24_0	273	0.22 (0.06; 0.60)
NEFA_24_1	273	0.13 (0.04; 0.31)
NEFA_24_2	273	0.03 (0.01; 0.06)
NEFA_24_4	273	0.03 (0.01; 0.07)
NEFA_24_5	273	0.03 (0.01; 0.09)
NEFA_26_0	273	0.24 (0.09; 0.50)
NEFA_26_1	273	0.14 (0.07; 0.28)
NEFA_26_2	273	0.08 (0.04; 0.15)
lyso.PC.a.C14.0	273	3.56 (1.27; 8.18)
lyso.PC.a.C16.0	273	102.64 (59.37; 178.23)
lyso.PC.a.C16.1	273	2.36 (1.03; 4.51)
lyso.PC.a.C18.0	273	25.41 (13.47; 43.36)
lyso.PC.a.C18.1	273	17.25 (9.73; 33.82)
lyso.PC.a.C18.2	273	23.72 (11.70; 47.06)

lyso.PC.a.C18.3	273	0.43 (0.14; 1.03)
lyso.PC.a.C20.3	273	2.88 (1.24; 4.67)
lyso.PC.a.C20.4	273	6.69 (3.75; 12.34)
lyso.PC.a.C20.5	273	0.39 (0.12; 0.86)
lyso.PC.a.C22.6	273	2.32 (1.24; 4.36)
lyso.PC.e.C16.0	273	0.98 (0.42; 1.96)
lyso.PC.e.C18.0	273	1.77 (0.82; 3.11)
lyso.PC.e.C18.1	273	0.28 (0.11; 0.51)
PC.aa.C30.0	273	5.36 (2.31; 12.44)
PC.aa.C30.3	273	0.22 (0.08; 0.46)
PC.aa.C32.0	273	14.18 (9.06; 23.94)
PC.aa.C32.1	273	16.54 (6.56; 36.23)
PC.aa.C32.2	273	5.50 (1.80; 12.52)
PC.aa.C32.3	273	0.45 (0.22; 0.85)
PC.aa.C34.1	273	229.12 (110.53; 415.01)
PC.aa.C34.2	273	453.93 (246.31; 772.54)
PC.aa.C34.3	273	18.45 (8.23; 37.10)
PC.aa.C34.4	273	2.53 (0.90; 5.26)
PC.aa.C34.5	273	0.22 (0.08; 0.53)
PC.aa.C36.0	273	1.78 (0.79; 3.38)
PC.aa.C36.1	273	42.97 (22.62; 81.48)
PC.aa.C36.2	273	216.65 (119.57; 366.21)
PC.aa.C36.3	273	188.68 (93.02; 342.05)
PC.aa.C36.4	273	247.59 (130.55; 437.78)
PC.aa.C36.5	273	21.22 (7.83; 47.53)
PC.aa.C36.6	273	1.26 (0.50; 2.79)
PC.aa.C38.0	273	3.83 (1.98; 6.85)
PC.aa.C38.2	273	7.36 (3.45; 13.60)
PC.aa.C38.3	273	56.93 (28.71; 113.17)
PC.aa.C38.4	273	117.62 (69.71; 198.67)
PC.aa.C38.5	273	57.50 (29.75; 100.16)
PC.aa.C38.6	273	129.42 (68.27; 234.34)
PC.aa.C40.0	273	1.35 (0.54; 2.51)
PC.aa.C40.1	273	0.54 (0.26; 1.25)
PC.aa.C40.2	273	0.35 (0.16; 0.72)
PC.aa.C40.3	273	0.59 (0.27; 1.21)
PC.aa.C40.4	273	4.20 (2.33; 8.59)
PC.aa.C40.5	273	12.48 (6.99; 24.06)
PC.aa.C40.6	273	40.09 (22.30; 68.76)
PC.aa.C42.0	273	0.96 (0.55; 1.91)
PC.aa.C42.5	273	0.60 (0.33; 1.05)
PC.aa.C43.6	273	3.35 (2.15; 6.17)
PC.aa.C44.12	273	0.77 (0.41; 1.48)
PC.ae.C30.0	273	0.45 (0.20; 0.88)
PC.ae.C32.0	273	3.67 (2.29; 6.68)
PC.ae.C32.1	273	2.70 (1.59; 4.73)
PC.ae.C32.2	273	0.63 (0.34; 1.25)
PC.ae.C34.0	273	1.42 (0.76; 2.63)
PC.ae.C34.1	273	9.59 (5.02; 17.09)
PC.ae.C34.2	273	10.48 (5.29; 18.38)
PC.ae.C34.3	273	7.75 (3.71; 15.52)
PC.ae.C34.4	273	0.30 (0.12; 0.60)
PC.ae.C36.0	273	0.77 (0.47; 1.40)
PC.ae.C36.1	273	5.51 (2.61; 9.48)
PC.ae.C36.2	273	14.16 (7.28; 24.53)
PC.ae.C36.3	273	8.71 (4.35; 16.09)
PC.ae.C36.4	273	17.21 (9.84; 30.33)
PC.ae.C36.5	273	11.21 (6.00; 20.25)
PC.ae.C38.0	273	2.63 (1.20; 5.57)
PC.ae.C38.2	273	1.96 (0.87; 3.18)
PC.ae.C38.3	273	4.66 (2.31; 8.17)
PC.ae.C38.4	273	13.88 (8.27; 22.73)

PC.ae.C38.5	273	18.19 (10.27; 32.08)
PC.ae.C38.6	273	8.06 (4.12; 14.23)
PC.ae.C40.0	273	18.79 (9.22; 36.26)
PC.ae.C40.1	273	1.66 (0.74; 2.85)
PC.ae.C40.2	273	1.67 (0.69; 3.16)
PC.ae.C40.3	273	1.01 (0.50; 2.08)
PC.ae.C40.4	273	2.90 (1.74; 5.04)
PC.ae.C40.5	273	3.93 (2.20; 6.39)
PC.ae.C40.6	273	5.46 (3.12; 9.15)
PC.ae.C42.1	273	0.46 (0.23; 0.93)
PC.ae.C42.3	273	0.94 (0.42; 1.78)
PC.ae.C42.4	273	1.41 (0.75; 2.57)
PC.ae.C42.5	273	3.26 (1.67; 5.88)
PC.ae.C42.6	273	2.11 (1.18; 3.75)
SM.a.C30.1	273	0.50 (0.23; 0.91)
SM.a.C32.1	273	9.09 (5.43; 14.17)
SM.a.C32.2	273	0.97 (0.54; 1.68)
SM.a.C33.1	273	6.48 (3.83; 10.55)
SM.a.C34.1	273	110.41 (73.33; 170.18)
SM.a.C34.2	273	19.41 (11.24; 31.09)
SM.a.C35.0	273	0.76 (0.39; 1.31)
SM.a.C35.1	273	3.34 (2.07; 5.60)
SM.a.C36.1	273	24.02 (15.24; 38.86)
SM.a.C36.2	273	12.57 (7.02; 21.21)
SM.a.C36.3	273	1.09 (0.51; 2.05)
SM.a.C37.1	273	2.13 (1.06; 4.00)
SM.a.C38.2	273	21.52 (10.20; 38.02)
SM.a.C38.3	273	0.70 (0.30; 1.40)
SM.a.C39.1	273	5.80 (3.00; 9.50)
SM.a.C39.2	273	1.89 (0.94; 2.96)
SM.a.C40.2	273	34.01 (18.57; 56.83)
SM.a.C40.5	273	0.89 (0.34; 2.05)
SM.a.C41.1	273	16.57 (9.62; 26.61)
SM.a.C41.2	273	14.12 (8.32; 22.03)
SM.a.C42.1	273	24.15 (15.89; 42.61)
SM.a.C42.2	273	65.72 (38.96; 104.89)
SM.a.C42.3	273	28.59 (16.53; 49.08)
SM.a.C42.4	273	8.65 (4.64; 15.74)
SM.a.C42.6	273	5.16 (2.55; 9.52)
SM.a.C43.1	273	1.39 (0.88; 2.54)
SM.a.C43.2	273	2.74 (1.58; 5.37)
SM.a.C44.6	273	1.94 (1.06; 3.27)
SM.e.C36.2	273	0.46 (0.26; 0.82)
SM.e.C38.3	273	0.33 (0.12; 0.63)
SM.e.C40.5	273	0.37 (0.17; 0.71)
Carn	273	25.30 (14.03; 38.19)
Carn.a.C10.0	273	0.14 (0.05; 0.43)
Carn.a.C10.1	273	0.10 (0.04; 0.19)
Carn.a.C12.0	273	0.09 (0.04; 0.17)
Carn.a.C14.1	273	0.05 (0.02; 0.12)
Carn.a.C14.2	273	0.03 (0.01; 0.07)
Carn.a.C15.0	273	0.04 (0.02; 0.09)
Carn.a.C16.0	273	0.14 (0.07; 0.31)
Carn.a.C16.0.Oxo	273	0.02 (0.01; 0.05)
Carn.a.C16.1	273	0.08 (0.04; 0.18)
Carn.a.C16.2	273	0.03 (0.01; 0.08)
Carn.a.C18.0	273	0.10 (0.05; 0.20)
Carn.a.C18.1	273	0.10 (0.04; 0.18)
Carn.a.C18.2	273	0.05 (0.03; 0.10)
Carn.a.C18.2.OH	273	0.03 (0.01; 0.05)
Carn.a.C2.0	273	2.78 (1.61; 5.42)
Carn.a.C20.0	273	0.04 (0.02; 0.07)

Carn.a.C20.1	273	0.00 (0.00; 0.00)
Carn.a.C20.3	273	0.06 (0.02; 0.12)
Carn.a.C20.4	273	0.01 (0.00; 0.01)
Carn.a.C3.0	273	0.28 (0.16; 0.48)
Carn.a.C3.0.DC	273	0.06 (0.03; 0.11)
Carn.a.C4.0	273	0.18 (0.08; 0.38)
Carn.a.C5.0	273	0.13 (0.07; 0.25)
Carn.a.C6.0	273	0.03 (0.01; 0.06)
Carn.a.C6.0.OH	273	0.04 (0.02; 0.07)
Carn.a.C8.0	273	0.08 (0.02; 0.23)
Carn.a.C8.1	273	0.06 (0.03; 0.13)
Carn.a.C9.0	273	0.03 (0.01; 0.07)
